# Rapid Contraceptive Uptake and Changing Method Mix With High Use of Long-Acting Reversible Contraceptives in Crisis-Affected Populations in Chad and the Democratic Republic of the Congo

**DOI:** 10.9745/GHSP-D-15-00315

**Published:** 2016-08-11

**Authors:** Jesse Rattan, Elizabeth Noznesky, Dora Ward Curry, Christine Galavotti, Shuyuan Hwang, Mariela Rodriguez

**Affiliations:** a CARE, Atlanta, GA, USA; b Independent consultant

## Abstract

The global health community has recognized that expanding the contraceptive method mix is a programmatic imperative since (1) one-third of unintended pregnancies are due to method failure or discontinuation, and (2) the addition of a new method to the existing mix tends to increase total contraceptive use. Since July 2011, CARE has been implementing the Supporting Access to Family Planning and Post-Abortion Care (SAFPAC) initiative to increase the availability, quality, and use of contraception, with a particular focus on highly effective and long-acting reversible methods—intrauterine devices (IUDs) and implants—in crisis-affected settings in Chad and the Democratic Republic of the Congo (DRC). This initiative supports government health systems at primary and referral levels to provide a wide range of contraceptive services to people affected by conflict and/or displacement. Before the initiative, long-acting reversible methods were either unknown or unavailable in the intervention areas. However, as soon as trained providers were in place, we noted a dramatic and sustained increase in new users of all contraceptive methods, especially implants, with total new clients reaching 82,855, or 32% of the estimated number of women of reproductive age in the respective catchment areas in both countries, at the end of the fourth year. Demand for implants was very strong in the first 6 months after provider training. During this time, implants consistently accounted for more than 50% of the method mix, reaching as high as 89% in Chad and 74% in DRC. To ensure that all clients were getting the contraceptive method of their choice, we conducted a series of discussions and sought feedback from different stakeholders in order to modify program strategies. Key program modifications included more focused communication in mass media, community, and interpersonal channels about the benefits of IUDs while reinforcing the wide range of methods available and refresher training for providers on how to insert IUDs to strengthen their competence and confidence. Over time, we noted a gradual redistribution of the method mix in parallel with vigorous continued family planning uptake. This experience suggests that analyzing method mix can be helpful for designing program strategies and that expanding method choice can accelerate satisfying demand, especially in environments with high unmet need for contraception.

## Abstract (precis)

Offering a broad choice of contraceptives can rapidly expand use in crisis-affected settings, particularly when the choice includes long-acting reversible contraceptives (LARCs). Over 5 years, the governments of Chad and the Democratic Republic of the Congo, with support from an NGO, provided nearly 85,000 new clients with contraceptives. LARC users, which included an increasing number of IUD users, accounted for 73%.

## Teaser

Offering a broad choice of contraceptives can rapidly expand use in crisis-affected settings, particularly when the choice includes long-acting reversible contraceptives (LARCs). Over 5 years, the governments of Chad and the Democratic Republic of the Congo, with support from an NGO, provided nearly 85,000 new clients with contraceptives. LARC users, which included an increasing number of IUD users, accounted for 73% of the new clients.

## BACKGROUND

Globally, approximately 225 million women have an unmet need for modern contraception—meaning that there are 225 million women who want to avoid pregnancy but are not currently using a contraceptive method.^1^^,^^2^ This global number obscures substantial geographical differences; 73% of women with unmet need live in the world’s poorest countries, with the majority living in Asia and sub-Saharan Africa. The proportion of women with unmet need in Central and West Africa is the highest in the world, at 81% and 74%, respectively.^2^ This high unmet need has serious public health consequences that contribute to high maternal mortality ratios. They include early, late, and poorly spaced pregnancies, pregnancy complications, and unsafe abortion.

The challenges of high unmet need and unintended pregnancy (and the attendant high rates of maternal death and unsafe abortion) in sub-Saharan Africa are not easily solved, but a necessary first step is to attract new users into family planning programs.^3^ These programs must reach women and girls who do not currently have access to services, while simultaneously increasing access of all users to the full range of contraceptive methods, especially highly effective and easy-to-use methods. Expanding access to a broad method mix has been recognized as a programmatic imperative.^4^ Lack of choice is associated with method discontinuation.^5^ Bradley and colleagues suggest that method failure is responsible for almost one-third of unintended pregnancies.^6^ Other research suggests that more than half of recent unwanted births in 14 countries with Demographic and Health Survey (DHS) data followed method failure or discontinuation.^7^ Furthermore, the addition of a new method to the existing mix tends to increase total contraceptive use.^4^

Since 2011, CARE has been implementing the Supporting Access to Family Planning and Post-Abortion Care (SAFPAC) initiative to increase the availability, quality, awareness, and use of contraception in crisis-affected settings in southern Chad and eastern Democratic Republic of the Congo (DRC). The initiative focuses on improving voluntary access to highly effective, easy-to-use long-acting reversible contraceptives (LARCs)—intrauterine devices (IUDs) and implants. The SAFPAC initiative supports government health systems that are chronically weak or unable to meet the demands of the populations they serve, especially communities experiencing chronic conflict or with large refugee and internally displaced populations. As of the end of November 2015, the project was supporting services in 19 facilities across 3 zones in DRC, reaching an estimated population of 505,447, of whom an estimated 126,362 were women of reproductive age. In Chad, the project supported 14 health facilities until January 2013, when it scaled up to support 21 facilities in 2 health districts in 2 regions, then reaching an estimated population of 543,480, of whom an estimated 135,870 were women of reproductive age. The initiative supports the entire range of methods included in the family planning programs in each country. CARE team staffing in both countries includes an overall coordinator for the zone or district, a clinical training coordinator, a clinical officer who leads supportive supervision, often in collaboration with government management, and a community mobilization and behavior change communication officer. Zonal and district teams also receive support from CARE staff members focused on procurement, supply chain management, and overall management. These teams work closely with government provincial, district, and zonal leadership and management teams, who co-lead all programmatic interventions.

The initiative focuses on improving access to highly effective, easy-to-use, long-acting reversible methods.

Our strategy includes strengthening the government health facilities, in particular, providing competency-based training with follow-up clinical assessment and coaching, ensuring a continuous supply of contraceptives and the medical supplies and equipment required to deliver them in accordance with international norms and standards, and conducting facility supervision on a regular basis in partnership with district and zonal health management staff. A key element of supportive supervision activities and a major part of the program overall is collecting monitoring data every month from each facility on the number of clients and the type of contraception they choose.

We also support competency-based training, coaching, and supervision for health care providers—nurses, midwives, and doctors—in government-managed facilities on 3 clinical skills: insertion and removal of IUDs and implants, postabortion care, and in-depth family planning counseling based on the Population Council’s “Balanced Counseling Strategy Plus Toolkit.”^8^ We do not train private providers in these countries. In DRC and Chad, SAFPAC-supported services, including long-acting methods, are offered during clinic hours (generally 8 am to about 1 pm) and do not include mobile clinics or special events or contraceptive/health days outside normal facility hours. In Chad, family planning services are technically free, but the client is often asked for an informal fee. In DRC, health workers receive a modest wage drawn from the national system of cost-recovery, meaning that clients are charged for certain services, including contraception. However, the SAFPAC approach requires contraceptive services to be provided free of charge. To manage this tension, CARE developed detailed agreements with government district and facility staff in Chad for the duration of the period discussed in this article, and in DRC in the second half of the program. The successful performance of these agreements is measured by the availability and high quality of free family planning services that *include all methods*, as well as the collection of monitoring data in addition to data already collected as part of the usual scope of providers’ work. We intentionally and explicitly do not set any contraceptive targets or offer incentives connected to numbers of clients or specific methods.

Successful performance is measured by the availability and high quality of free family planning services that include all methods.

Because raising people’s basic awareness of new and underused methods is a necessary (if insufficient) first step, we also work at the community level. We work with community health workers (CHWs) in DRC (there is no formal CHW cadre in Chad), local community-based organizations, theater groups, and radio stations to increase understanding and awareness of all contraceptive methods and especially less familiar long-acting methods. In addition, an important part of the SAFPAC community strategy is engaging and inspiring the whole community to consider the benefits and opportunities inherent not only in planning and spacing births and decreasing unintended pregnancies, but also in clearing the path for women to use services. Each country program carries out a context-specific strategy to develop champions among influential men and women in the community, most often religious leaders, municipal authorities, and leaders of women’s associations in Chad and *misadis*, or trusted, supportive, and influential women, in DRC. We use participatory learning and action methods in collaboration with these leaders to engage communities in dialogues on the sensitive topics of contraception and women’s right to manage their fertility. We describe our programmatic interventions in more detail elsewhere.^9^

This article focuses on our work in Chad and DRC, where maternal mortality ratios at the beginning of our program were especially high (1,099 per 100,000 live births in Chad^10^ and 846 per 100,000 live births in DRC^11^). Since one-third of all maternal deaths might be prevented by satisfying unmet need for contraception,^12^ we can assume that very low levels of use of modern contraceptive methods contribute substantially to these high maternal mortality ratios, in part by fueling high rates of unsafe abortion.

We looked at DHS data before starting our program. The 2004 DHS for Chad found that only 2.5% of all women of reproductive age currently used a contraceptive method.^10^ Slightly more than half of these women reported using either periodic abstinence (0.9%) or condoms (0.4%). These 2 methods have some of the highest “actual use” failure rates of either traditional or modern methods. Of the 1.5% of women using a modern method, short-acting methods dominated the mix, with long-acting reversible and permanent methods comprising only 7.5% of the modern method mix. Although total unmet need for contraception was reported as 18.3% of all women of reproductive age surveyed, it is likely to be much higher, since the need of women using traditional methods for more effective methods was not expressed.^2^

In DRC in 2007, the DHS reported that 20% of all women of reproductive age currently used a contraceptive method.^11^ Among contraceptive users, two-thirds (13.4% of all women of reproductive age) reported using a traditional method, whereas only one-third (6.7% of all women of reproductive age) reported using a modern method. Among those using modern methods, condoms comprised 72% of the method mix. Unmet need in DRC was 24%. As in Chad, this is most likely an underestimate. Less than half (48%) of all sexually active unmarried women surveyed had ever used a contraceptive method. This and the use of methods with high failure rates suggest a much greater need for highly effective contraception.

In Chad, only 1.5% of women of reproductive age used a modern contraceptive method in 2004. In the DRC, 6.7% used a modern method in 2007.

Before the start of SAFPAC, family planning services were available, in theory, through the government health facilities in both Chad and DRC. In practice, however, people rarely used these services, especially outside of hospitals and urban areas. Furthermore, in the intervention zones where the SAFPAC initiative started in Chad and DRC in mid-2011, these services were largely limited to short-acting methods. Our pre-program assessments identified a number of reasons for this. In Chad, national guidelines authorized only doctors based in hospitals to provide IUDs and implants, and, in both Chad and DRC, few providers had been trained to provide either family planning counseling or LARC services. As a result, providers lacked both the skill and confidence to deliver these services. Even when trained providers were available, extremely weak contraceptive supply chains led to chronic stock-outs, which meant that most of the time there were few, if any, methods available. The cost of services was also an inhibiting factor. Although both contraceptives and consultations are supposed to be free in accordance with Chad’s national reproductive health policy, providers often charged informal fees for their services in order to supplement meager and irregularly paid salaries. In DRC, facilities are permitted to charge user fees for certain services, including contraceptive services, in order to recover their cost of operation. Demand for services was further weakened by the lack of good information about contraceptive services, especially LARCs, combined with conservative social norms that stigmatize the use of contraception. These factors are challenges in stable populations. In the crisis-affected areas where CARE works in DRC and Chad, these barriers are magnified for displaced people and refugees who may not be aware of the different methods available to them, may not know where to get them, or, because of insecurity, may be unable to consistently access these services, even though studies show that they have a need for spacing and limiting births, as in any population.^13^

In the first 3 years of programming in Chad and DRC, we noted a dramatic and sustained increase in new users of all contraceptive methods, especially implants. In the first 6 months following provider training, demand for implants was so strong that implants consistently comprised more than 50% of the method mix each month (reaching as high as 90% in Chad and 74% in DRC). While there is no correct mix of contraceptive methods, women must be able to access and freely choose from a wide range of contraceptives. A skewed method mix, defined as more than 50% of users relying on a single method, can indicate lack of choice.^14^ We expected a robust uptake of methods previously unavailable, as a result of pent-up demand. Nevertheless, 6 months after the initial trainings on LARCs, we decided to examine method mix patterns more closely and to investigate the dynamics driving choice of methods, especially implants and IUDs. We wanted to ensure that our program was promoting full contraceptive method choice and client-centered services.

We noted a dramatic and sustained increase in new users of all contraceptive methods, especially implants.

## METHODS

We used both quantitative and qualitative methods to examine contraceptive use patterns and factors contributing to the observed method mix.

### Quantitative Data

Beginning in January 2012, we reviewed monthly routine service delivery data collected from 33 supported health facilities (14 in Chad and 19 in DRC), reaching a combined population of 1,053,927 in Logone Oriental and Moyen Chari provinces in southern Chad and in Maniema and North Kivu provinces in DRC between June 2011 and June 2015. These data include the number of new contraceptive users by method, month, and facility. We define a new family planning user as a client who is starting a modern method, switching from a traditional or a different modern method to a new modern method, or who is restarting a modern method after 6 months of nonuse. Our programmatic goal was to increase access to all methods, with attention focused on increasing the availability of little-known, highly effective long-acting reversible methods. We collected data on 6 modern contraceptive methods: oral contraceptive pills (OCPs), injectables, implants, IUDs, tubal ligation, and no-scalpel vasectomy. We did not count traditional methods. We also did not track methods with typical-use effectiveness less than 90%.^15^ Thus, while we ensured that condoms were widely available and offered, we did not track new users of condoms. The Lactational Amenorrhea Method and Standard Days Method/CycleBeads are not consistently offered in the facilities that we supported, and so we did not monitor their use. This means that our reported method mix is not comparable to the broader contraceptive method mix studied in many national population surveys. However, it is possible to see trends in the data from each health facility.

Without training, coaching, guidance, simple tools, or positive support for data collection, the quality of data from public health facilities in eastern DRC and southern Chad can be very poor. One of the SAFPAC initiative’s core elements was developing, in collaboration with government providers and managers, a robust system to track basic monitoring information (see the supplementary material for the monitoring and evaluation data flowchart). Service delivery data were drawn from existing government tools, such as registers, as well as new tools that we designed to fill the need for simple tools to track contraceptive users as well as for our specific monitoring needs for this program. CARE staff and providers collected de-identified user information, including choice of method, on a hard-copy monthly data collection form designed by CARE. Each district team entered this information by facility and month into a Microsoft Excel spreadsheet. District information was then rolled up to the country level, where SAFPAC staff members compiled and reviewed the data as a Microsoft Excel data set and then sent it to the global program team for compilation and analysis.

Data quality was spotty in the initial phase of programming, as CARE facility and district staff learned how to use the new indicators, definitions, and data collection tools through a series of intensive 5-day trainings. One of the key factors in building data quality was consistent, regular supportive supervision by SAFPAC staff and government district and zonal staff. SAFPAC staff conducted regular data quality checks during monthly supervisory visits by cross-checking data in different records/datasheets. The global team was not the only group to analyze data; facility, district, and CARE staff members met during these visits to review, double-check, analyze, and make rapid action plans based on trends in the data. Teams at multiple levels also reviewed the data for errors. The initiative further benefited from a third-party monitor who made annual visits to all project sites from 2012 through 2015 to review the overall progress of the initiative and, in particular, to review data quality, completeness, and use.

One of the key factors in building data quality was consistent, regular supportive supervision.

For this article, we analyzed data from the global aggregated datasheet for the period from June 2011 to November 2015. We created pivot tables and generated graphs and charts to describe trends in the percentage distribution of new users by method at national and subnational levels.

### Qualitative Data

We created a set of activities designed to assess communities’ and providers’ understanding of and attitudes toward various contraceptive methods. We integrated these activities into our participatory community reviews of facility data on trends in new users of contraception. We began our investigation by comparing the high uptake of implants to the relatively low uptake of IUDs. In Chad and DRC, during monthly and quarterly data review meetings with providers, health officials, community leaders, and community members, we explored reasons that implants were popular and IUDs were not. Data visualization was a core part of our participatory monitoring strategy and featured in the focused investigation of the unusual method mix. Facility staff members drew pie charts for contraceptive method mix and line graphs for trends over time on flip charts that were displayed in the health facility and modified monthly based on data drawn from the register and collected in a simple form. During community meetings, men and women leaders in the community would review the wall charts, and they updated and sometimes corrected them.

In Chad, for this focused investigation, we reviewed the hand-drawn flip chart with user data displayed in the form of pie charts and graphs, and then we conducted informal focus group discussions with 15 community members from each of the 14 facility catchment areas, totaling 210 male and female community leaders (including both users and nonusers of contraception among the women leaders). We also interviewed 60 providers in small, informal focus group discussions. In Chad, the basic questions that we asked were: What are the factors underlying the high uptake of implants? Do women prefer them and, if so, why? Do providers prefer them and why? What are the positive and negative aspects of implants and of IUDs? What could be some strategies to communicate more effectively with clients and the public about a full range of contraceptive methods, including IUDs?

In DRC, we employed CARE’s Social Analysis and Action approach,^16^ a participatory rapid appraisal technique, to conduct activities with women (both users and nonusers of family planning), adolescents (both in school and out of school), CHWs, health officials, and religious leaders (imams, pastors, and priests) to explore attitudes and beliefs concerning implants and IUDs in a nonjudgmental manner. The Social Analysis and Action approach is built on participatory learning and action tools and activities that help groups and individuals to raise and nonjudgmentally explore underlying factors related to power, gender, and culture that support or inhibit people’s access to health services. In DRC, 500 people participated in small-group discussions. This number was slightly higher than in Chad, because the DRC initiative supported 19 health facilities, thus serving more communities than in Chad (which supported 14 health facilities). CARE’s community engagement officers, who convened these discussions, asked questions similar to those asked in Chad: Why is the uptake of implants so high? Why do women choose implants more than any other method? What are the barriers to more women using IUDs? Do providers prefer implants to IUDs and, if so, why? Are there barriers to women’s access to a full range of contraceptive methods, especially IUDs and, if so, why? What are some strategies to ensure that women have access to a full range of modern contraception? Teams in both countries took notes in notebooks or on computers during the discussions, debriefed together after them, and proposed a set of solutions, including key messages, drawn from community and provider feedback and their own analysis. The Chad and DRC teams then further developed potential solutions with the SAFPAC global team, either face-to-face or virtually.

Discussion questions included: “Why do women choose implants more than any other method?” and “Do providers prefer implants to IUDs and, if so, why?”

Both quantitative and qualitative data were collected for the purpose of the continuous quality improvement of the project, not for the purpose of conducting systematic research in a strictly defined model. All data collection and analysis were conducted according to international principles of maintaining privacy and confidentiality of personal information.

## RESULTS

### Quantitative Data: Contraceptive Use and Method Mix Patterns

The SAFPAC initiative delivered family planning services to 82,855 people in Chad and DRC between June 2011 and November 2015. In Chad, we provided family planning services to 46,571 new users, for an average of 862 new users per month, reaching 34% of the eligible population (i.e., women of reproductive age). In DRC, we provided services to 36,284 new users, for an average of 685 new users per month, reaching 29% of the eligible population. In Chad, 55% (n = 25,610) of all new users received services from primary health centers at the community level. Some 17% (n = 7,942) received services from the district hospital, and the remaining 28% (n = 13,019) received them from the regional hospital (Table 1). In DRC, 61% (n = 22,103) of all new family planning users received services from primary health centers at the community level. Some 22% (n = 7,835) received services from the referral primary health center. The remaining 17% (n = 6,346) received services from the referral hospital for the health zone (Table 1).

**TABLE 1.** Number and Percentage of New Family Planning Users by Facility Type, Chad and DRC, June 2011 to November 2015

Health Facility Type	Chad No. (%)	DRC No. (%)
Primary health centers	25,610 (55)	22,103 (61)
District hospital^a^/referral primary health centers^b^	7,942 (17)	7,835 (22)
Regional hospital^a^/referral hospitals for the health zone^b^	13,019 (28)	6,346 (17)
**Total**	**46,571 (100)**	**36,284 (100)**

Abbreviation: DRC, Democratic Republic of the Congo.

a For Chad.

b For DRC.

**FIGURE 1.**
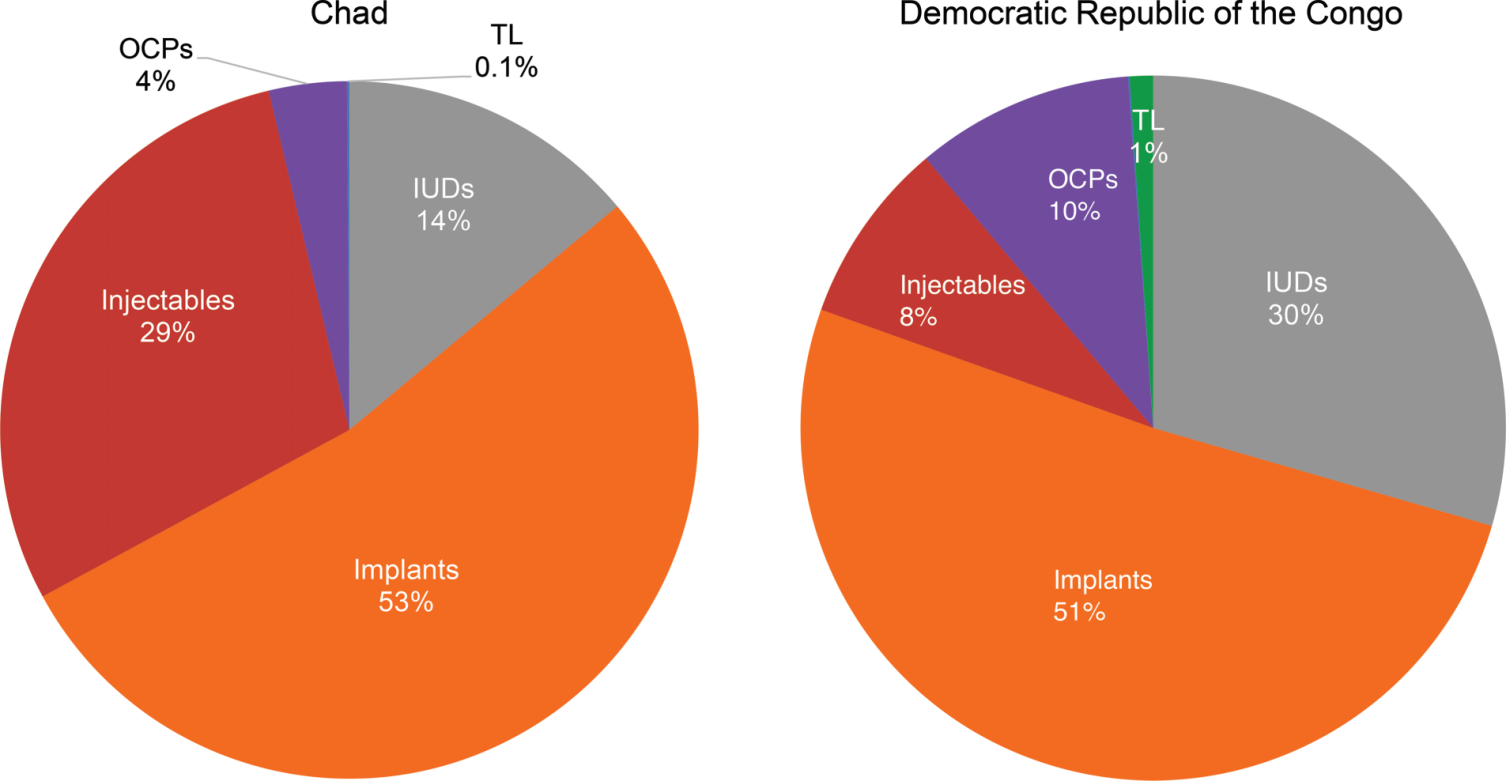
Contraceptive Method Mix Among New Family Planning Users in Program Areas in Chad^a^ and DRC, June 2011 to November 2015 Abbreviations: DRC, Democratic Republic of the Congo; IUDs, intrauterine devices; OCPs, oral contraceptive pills; TL, tubal ligation. ^a^ None of the family planning users in Chad chose no‐scalpel vasectomy.

In both countries, large numbers of people started using family planning methods, but the contraceptive method mix differed somewhat, even though the same methods were available in the intervention areas in both countries (Figure 1). In Chad, 53% (n = 24,753) of all new users chose implants; 29% (n = 13,601) chose injectables; 14% (n = 6,485) chose IUDs; 4% (n = 1,679) chose OCPs; <1% (n = 53) chose tubal ligation; and none chose no-scalpel vasectomy. In DRC, 51% (n = 18,495) of all new family planning users chose implants; 30% (n = 10,696) chose IUDs; 10% (n = 3,629) chose OCPs; 8% (n = 3,049) chose injectables; 1% (n = 388) chose tubal ligation; and less than 1% (n = 27) chose no-scalpel vasectomy.

In Chad, during this period primary health centers, district hospitals, and the regional hospital had similar contraceptive method mixes, with implants dominating, followed by injectables, IUDs, and OCPs, while a small number chose tubal ligation. Although primary health centers contributed only 55% of all new family planning users, they accounted for a disproportionate 65% of new OCP users and 60% of new IUD users. The district hospital, which contributed 17% of all new family planning users, accounted for two-thirds (66%) of the few tubal ligation clients. The regional hospital, which contributed 28% of all new family planning users, accounted for one-third (33%) of new users of injectables (Table 2).

**TABLE 2.** Source of Family Planning Service by Type of Method, Chad and DRC, June 2011 to November 2015

Method	Chad—No. (%)				DRC—No. (%)			
	Primary Health Centers	District Hospital	Regional Hospital	Total	Primary Health Centers	Referral Primary Health Centers	Referral Hospitals for the Health Zone	Total
IUDs	3,878 (59.8)	1,095 (16.9)	1,512 (23.3)	**6,485 (100.0)**	5,843 (54.6)	3,166 (29.6)	1,687 (15.8)	**10,696 (100.0)**
Implants	13,910 (56.2)	4,207 (17.0)	6,636 (26.8)	**24,753 (100.0)**	11,363 (61.4)	3,596 (19.4)	3,536 (19.1)	**18,495 (100.0)**
Injectables	6,723 (49.4)	2,376 (17.5)	4,502 (33.1)	**13,601 (100.0)**	2,189 (71.8)	447 (14.7)	413 (13.5)	**3,049 (100.0)**
OCPs	1,089 (64.9)	229 (13.6)	361 (21.5)	**1,679 (100.0)**	2,600 (71.6)	490 (13.5)	539 (14.9)	**3,629 (100.0)**
No-scalpel vasectomy	0 (0.0)	0 (0.0)	0 (0.0)	**0 (0.0)**	8 (29.6)	1 (3.7)	18 (66.7)	**27 (100.0)**
Tubal ligation	10 (18.9)	35 (66.0)	8 (15.1)	**53 (100.0)**	100 (25.8)	135 (34.8)	153 (39.4)	**388 (100.0)**
**All users**	**25,610 (55.0)**	**7,942 (17.1)**	**13,019 (28.0)**	**46,571 (100.0)**	**22,103 (60.9)**	**7,835 (21.6)**	**6,346 (17.5)**	**36,284 (100.0)**

Abbreviations: DRC, Democratic Republic of the Congo; IUDs, intrauterine devices; OCPs, oral contraceptive pills.

In DRC, during this period all 3 facility types (primary health centers, referral primary health centers, and referral hospitals for the health zone) had similar contraceptive method mixes, with implants dominating, followed by IUDs, injectables, OCPs, tubal ligation, and a few no-scalpel vasectomies. Although primary health centers contributed 61% of all new family planning users, they accounted for a disproportionately large share of new OCP users (72%) and injectables users (72%). The referral primary health centers, which contributed 22% of all new family planning users, had a disproportionately large share of tubal ligation clients (35%) and new IUD clients (30%), whereas the hospital, which contributed 17% of all new family planning users, accounted for a disproportionately large share of no-scalpel vasectomy clients (67%) and tubal ligation clients (39%) (Table 2).

**FIGURE 2.**
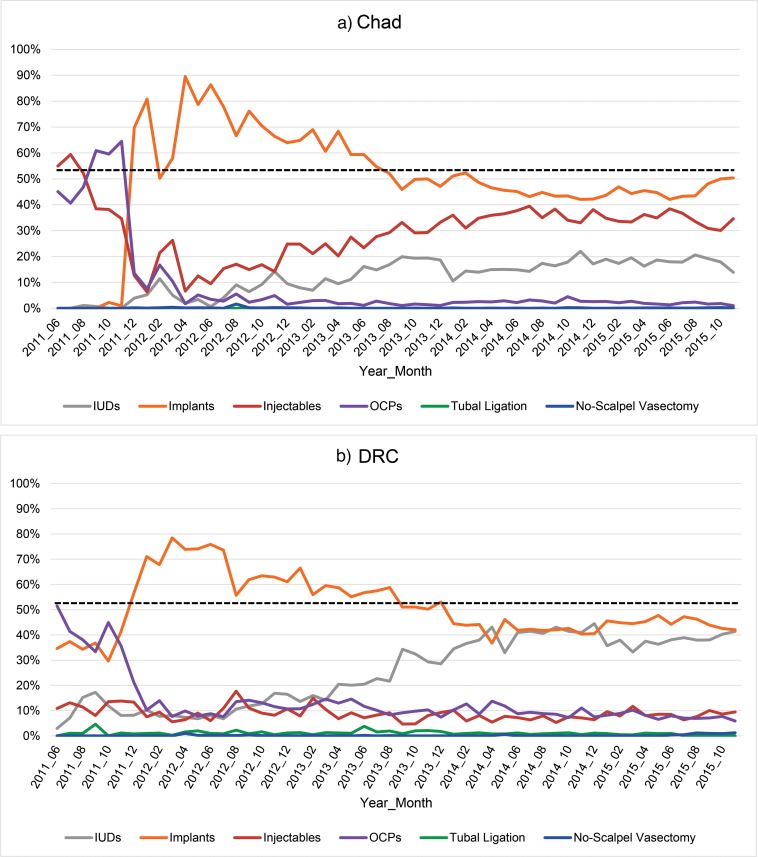
Monthly Trends in Uptake, as a Percentage of Total Uptake, of Different Contraceptive Methods Among New Family Planning Users in Program Areas, Chad (N=46,571) and DRC (N=36,284), June 2011 to November 2015 Abbreviations: DRC, Democratic Republic of the Congo; IUDs, intrauterine devices; OCPs, oral contraceptive pills.

However, these cumulative figures mask the fact that the contraceptive method mix changed dramatically in both countries over 4 years. Figure 2 shows the percentage change over time in the 2 countries for the 6 main methods we focused on—implants, IUDs, injectables, OCPs, tubal ligation, and no-scalpel vasectomy, even though the latter 2 methods were available in only a minority of facilities in both countries.

Both countries saw a sharp increase in the uptake of implants after providers were trained on long-acting reversible methods in November and December of 2011. Implants dominated the method mix for the next 5–6 months, displacing injectables in Chad and OCPs in DRC as the most popular method. In terms of percentage of the method mix, implants reached their peak in Chad in April 2012 at 90% and in DRC in March 2012 at 78%.

From this point onward, both countries saw a steady, gradual decline in the percentage of the mix attributable to implants, so that the method mix in both countries was more evenly distributed by the end of 2013. Since then, the total number of new users per month has remained stable or increased, and the method mix has remained balanced and fairly stable in both countries. In both countries, OCP use relative to other methods has decreased the most, and in DRC, IUD use has increased to more than 40% of the total method mix.

The method mix in both countries was more evenly distributed by the end of 2013 and has remained balanced and fairly stable since then.

### Qualitative Data: Factors Contributing to Early Contraceptive Use Patterns

Our interviews and discussions with providers and community members revealed several factors contributing to the high initial uptake of implants and the relatively low uptake of IUDs. These include both positive factors associated with implants and negative factors associated with IUDs.

#### Demand-Side Factors

- **Positive impressions of implants through word-of-mouth:** Implants created a buzz, and uptake was swift, as satisfied users talked about them. In Chad, many women reported choosing an implant because friends, family members, and neighbors had chosen implants and recommended them. In environments where there is little access to information through mass media, word-of-mouth and personal recommendations from trusted family and friends are very influential, as has been documented elsewhere.^17^^,^^18^
- **Branding of the implant:** In Chad, many women liked the name of the implant “Jadelle” because it sounded like a woman’s name and was easy to remember. The implant also acquired a nickname, *les deux batons,* or “the 2 sticks”—another indication that word-of-mouth from satisfied clients contributed to uptake. In Chad, providers and community members said it was difficult to communicate effectively about IUDs because of the challenge of translating “intrauterine device” into local languages.
- **Modesty/discretion:** Women liked implants because they last a long time, require only one trip to the health facility, and could be used without a partner’s knowledge. Furthermore, implants did not require women to undergo a pelvic exam, unlike IUDs (which share many of the same benefits). Some women were unaccustomed to disrobing at all during health center visits. In particular, they were quite reluctant to disrobe in front of a male provider for an IUD procedure. In Chad, some women reported that not only were pelvic exams intrusive and undesirable, but also that they felt shame about the condition of their undergarments and their personal hygiene.
- **Fear and misconceptions about the IUD:** In DRC and Chad, providers and clients both held misconceptions about the IUD—that it caused cancer and infertility, that the string can bother the male partner during intercourse, and that it can cause malformations in the fetus. In Chad, community leaders displayed the IUD in its plastic package, which included the insertion trocar, making the IUD look very long and unlikely to fit inside a woman’s body. In Chad, women also commented that they preferred the implant because after insertion it was palpable or visible under the skin, compared with the IUD, which could not be seen once inserted. This perception was linked to women’s fear that the IUD, once placed, could move around in the body and possibly disappear.
- **Novelty of implants:** While this concept is intertwined with positive impressions, advantageous characteristics, word-of-mouth, and pent-up demand, implants were a new and, therefore, novel product in the intervention areas. People were already familiar with short-acting methods and were minimally familiar with IUDs. We believe that we experienced an initial spike in demand due in part to the newness of the method, which may have heightened its appeal. Similar spikes in demand following the introduction of a new method were noted for the IUD and pill in national family planning programs in several Asian countries during the early 1970s^4^ as well as for injectables in sub-Saharan Africa in the 1990s and 2000s.^19^
- **Negative associations with IUDs:** In DRC, negative impressions of the IUD persisted in some areas where, before program implementation, clients had complications after providers without training in IUD insertion had placed IUDs poorly. Osei and colleagues have described similar dynamics in Ghana.^20^

We believe there was an initial spike in demand for implants because of the newness of the method, heightening its appeal.

Women liked implants because they last a long time, require only one trip to the health facility, and could be used without a partner’s knowledge.

#### Supply-Side Factors

- **Providers’ comfort with implant insertion:** Health care providers in most settings are under time pressure. Providers in our program areas stated that implants were relatively easier and faster to provide than IUDs, in some cases taking only 10 minutes for the actual insertion. As a result, providers favored implants among the LARC methods. This was especially important in a context in which providers feel compelled to offer services to the 20 to 30 clients who present each morning and yet must farm or work other jobs in the afternoons because their jobs as health care providers pay poorly and often irregularly.
- **More opportunity for practice with implants:** Because implants were so popular with clients, providers had much more opportunity to practice implant procedures than IUD placements during training and in their actual practice. Both feedback from providers and clinical skills assessments indicated that more providers were competent and confident in implant insertion/removal than in IUD insertion/removal, creating a reinforcing feedback loop of client and provider preference for this method.
- **Misconceptions about IUDs:** The fear of untreated sexually transmitted infections (STIs) leading to pelvic inflammatory disease caused a decline in IUD use in sub-Saharan Africa in the 2000s.^21^ Similarly, in our discussions many providers mentioned their concern that STIs, which they identify through syndromic management, contraindicate placement of an IUD, and inconsistent availability of antibiotics made it hard for them to treat STIs. As a result, providers were hesitant to provide IUDs to women who might have an STI but where the facility had no antibiotics to treat the STI, resulting in missed opportunities to provide the method. In Chad, some providers and many community leaders also erroneously believed that IUD use actually causes STIs.

## PROGRAM REVISED IN RESPONSE TO FEEDBACK

Based on our analysis of the quantitative data and the results of the informal, participatory qualitative investigation that we conducted, we rapidly modified our programmatic activities. A summary of our modified strategies and activities follows.

### Demand-Side Solutions

- In Chad, we worked with committees of religious leaders to better communicate the positive characteristics of IUDs while simultaneously reiterating that a broad range of methods was available at health centers. For example, one message pointed out the advantage of receiving a pelvic exam during IUD insertion, which could be used to conduct additional screening for STIs.
- Because the term “intrauterine device” was so difficult to translate, the Chad team discovered a simple local name built upon the widely used term for implants—“the 2 sticks”—and named the IUD “1 stick.” This name was well accepted and was later adopted in the DRC program as well.
- In DRC, we placed greater emphasis on IUDs in all our mass media and community mobilization work using radio, theater groups, and group discussions (including satisfied clients) while at the same time discussing a broad range of methods. We pointed out the benefits of IUDs, including immediate return to fertility, which was often mentioned in discussions as a positive characteristic of the IUD. We also addressed the myths and misconceptions concerning IUDs.

Because the term intrauterine device was difficult to translate, the Chad program name the IUD “1 stick,” building on the local and widely used term for implants (“2 sticks”).

### Supply-Side Solutions

- We introduced and trained providers on the Population Council’s “Balanced Counseling Strategy Plus Toolkit.”^8^ This counseling approach encourages the provider to focus first on what the client wants. It also includes visual job aids—small laminated cards that have pictures of each method on the front for the client and efficacy and side effects information on the back, which cue the provider to share this information with the client. As a result, clients received better counseling about the pros and cons of each method, including side effects. We also reinforced counseling that dispelled local rumors such as the IUD causing cancer, the string bothering the partner during sex, and the possibility of a baby being born holding the IUD.
- Since the low level of demand for IUDs led to fewer chances for providers to practice, thus contributing to providers’ lack of confidence in providing this method, we conducted refresher training, using anatomic models, to strengthen provider competence and confidence. We focused on making sure providers understood the medical eligibility criteria for IUDs (and implants), which, for example, allow nulliparous women to use either method.
- We trained providers on syndromic management of STIs and ensured a stock of antibiotics in all clinics. In keeping with current World Health Organization (WHO) guidelines, we made a distinction between current purulent cervicitis (chlamydial infection and gonorrhea) and other STIs and vaginitis and the timing of IUD insertions. All cases of purulent cervical discharge (WHO Medical Eligibility Criteria category 4) needed to be treated first, and the condition resolved, before an IUD could be inserted. If a woman already had an IUD in place, the provider treated the condition without the need to remove the IUD, if the client wanted to continue to use the method (WHO Medical Eligibility Criteria category 2). For other STIs, a woman could have an IUD inserted (WHO Medical Eligibility Criteria category 2), while simultaneously being treated for bacterial vaginosis and other non-purulent STIs.
- In DRC, we developed agreements with facility teams to compensate providers more fairly for the extra time and complexity of offering an expanded range of free services, as well as the extra effort of data collection.

**FIGURE 3.**
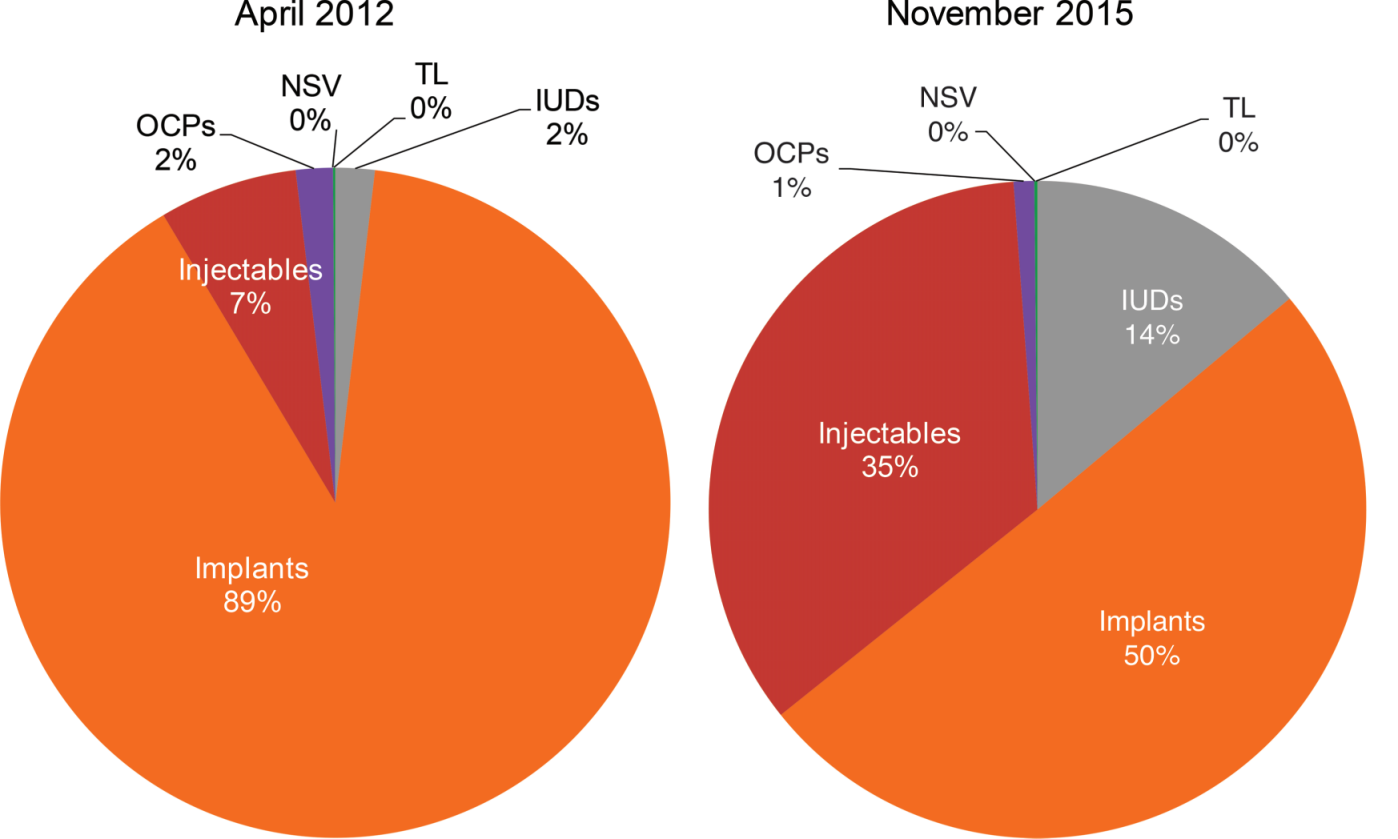
Changes in the Contraceptive Method Mix Among New Family Planning Users in Program Areas, Chad, April 2012 Compared With November 2015

**Figure 4.**
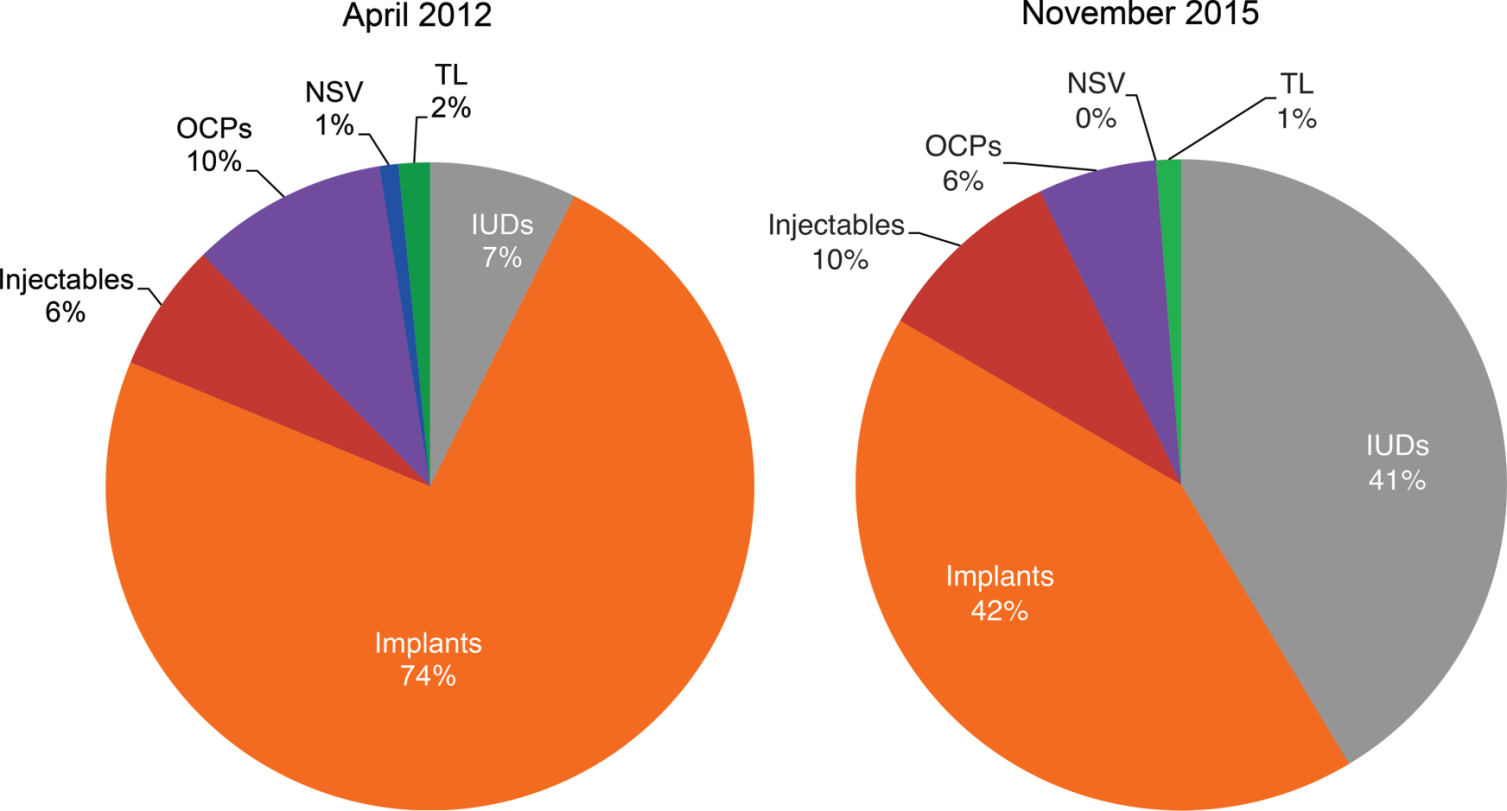
Changes in the Contraceptive Method Mix Among New Family Planning Users in Program Areas, DRC, April 2012 Compared With November 2015 Abbreviations: DRC, Democratic Republic of the Congo; IUDs, intrauterine devices; NSV, no‐scalpel vasectomy; OCPs, oral contraceptive pills; TL, tubal ligation.

We noted a gradual shift in method mix over time, culminating in a more evenly distributed method mix by November 2015. For example, in April 2012 in Chad, implants accounted for 89% of the method mix, injectables 7%, and IUDs 2%. In November 2015, the method mix shifted to where implants accounted for only 50% of the mix, and injectables and IUDs increased to 35% and 14% of the mix, respectively (Figure 3). Similarly, in April 2012 in DRC, implants accounted for 74% of the method mix, IUDs 7%, and injectables 6%. In November 2015, implants and IUDs accounted for nearly equal shares of the method mix (42% and 41%, respectively), while injectables accounted for 10% of the mix (Figure 4). We believe our adjustments in programming influenced this change.

## DISCUSSION

There has been a resurgence of global attention to meeting the large and growing need for contraception, especially in developing countries, through initiatives such as Family Planning 2020 (FP2020) and the United Nations Every Woman Every Child initiative. Importantly, the global conversation has included discussions about quality of care, highlighting choice and voluntarism as key components of quality programming that respects and fulfills a woman’s right to receive the contraceptive method of her choice.^22^

Our analysis contributes to this discussion by describing trends in method mix over time in 2 countries—Chad and DRC—where we implemented a focused set of interventions to improve access to and use of modern contraception, especially effective, long-acting reversible methods. Noting an early surge in use of implants, we explored factors that might underlie these trends. This analysis revealed a number of both demand- and supply-side factors contributing to these patterns. As a result, we made several changes to further improve programming and to ensure that women were being supported in making contraceptive decisions in the context of full choice.

As our results demonstrate, SAFPAC succeeded in catalyzing large increases in the numbers of new users of contraception in both Chad and DRC. After we conducted the first competency-based training of providers on clinical skills for IUD and implant insertion/removal in late November and early December 2011, we saw a dramatic increase in the number of new contraceptive users, especially those choosing implants. In Chad, demand for implants was so unexpectedly strong that health facilities experienced temporary stock-outs of this method in January and February 2012, which we resolved by late March. In April 2012, the percentage distribution of implants in the method mix reached its apex of 90%. We also saw a rapid uptake of implants in DRC, where implants reached their high point of 76% of the method mix in March 2012. After ruling out data collection errors as a source of substantial overcounting, we held discussions between July and December of 2012 with both providers and community members to help us better understand these trends. These discussions confirmed that there was no outright coercion or other major barriers to women either receiving the method they came in to get or being satisfied with the one they left with.

During community conversations, clients spontaneously described many of the advantages of both implants and IUDs, but uptake of IUDs remained low. Accordingly, we placed particular emphasis on leveling the playing field for IUDs in relationship to implants (and also popular short-acting methods). We thought that, in addition to strengthening providers’ clinical skills, a major strategy should be to communicate the characteristics of IUDs more effectively both during client-provider interactions and in community messaging.

We placed particular emphasis on leveling the playing field for IUDs in relationship to implants.

As the data attest, the method mix gradually adjusted, including a steady relative decrease in implants and a steady relative increase in IUDs (as well as injectables in Chad) from mid-2012 to 2015. This shift coincided with our programmatic modifications designed to equalize access to all methods, in alignment with Hardee and colleagues’ broad definition of what constitutes access in family planning programs.^22^ This more evenly distributed method mix continues in our current programming. The high number of new users that we followed (more than 80,000), the consistency of trends, and the size of the observed change suggest an association between our program modifications and the shift in the method mix.

Finally, recent measures of the modern contraceptive prevalence rate (mCPR) by the national DHS survey in Chad, published in 2015, and in DRC, published in 2014, suggest that this expanded access to a broad range of methods, and in particular to LARCs, has contributed to an overall increase in contraceptive use in the rural and crisis-affected provinces and regions where CARE works. For example, in DRC the national mCPR in 2014 was 7.8%, compared with 8.2% in remote and rural Maniema province, where CARE is one of the few partners supporting family planning services generally and the only partner supporting LARCs. In North Kivu province, where CARE and 2 other NGO partners are supporting a modest number of health zones to improve family planning in this large and populous province, the mCPR is 11.6%, surpassed only by Kinshasa (19.0%) and Bas-Congo (17.2%), which are urban and peri-urban provinces.^11^ In Chad, for most of the period covered, CARE was the only partner supporting the government to provide a full range of contraceptive services, including LARCs, in the Moyen Chari and Logone Oriental regions. The mCPR in these 2 regions is 11.4% and 11.3%, respectively, more than twice the national average of 5%.^23^ The 2 regions bordering Moyen Chari and Logone Oriental, namely Mandoul and Logone Occidental, have mCPRs of 12.6% and 10.5%,^23^ respectively, again with essentially no external support except from CARE.

### Limitations

Careful review of our routine monitoring data, both through random chart and register reviews and the cleaning and reviewing of the spreadsheets at the country office and the global level, revealed no obvious problems with data quality that could affect these trends. Our participatory discussions with community stakeholders and providers followed good programmatic practices, including consistent questions across geographic areas in each country, note-taking, and then analysis by the project team. However, these efforts should be viewed as monitoring activities, intended to inform and improve a dynamic NGO program, rather than as formal research. For example, the absence of a control group or cross-sectional baseline and endline surveys, which could show changes in the contraceptive prevalence rate specific to the CARE-supported facility catchment areas, limit our interpretation and also wider generalizability.

## CONCLUSION

When the SAFPAC initiative began in Chad and DRC, implants were a relatively unknown method, particularly in Chad. Rapid uptake followed their introduction in Chad and to a lesser extent in DRC. It is remarkable that large numbers of women chose these new and unknown methods so quickly and vigorously in the early and later phases of the project. This is not a new phenomenon, however. The introduction of injectables, and more recently implants, in sub-Saharan African countries over the past decade led to a similar dramatic increase in numbers of new users, and other programs providing intensive support for the introduction of LARCs or other neglected methods have had similar results.^24^^,^^25^ The introduction of a new method in a country often changes the method mix, either by increasing the number of new users or by inducing people to switch methods. In the latter instance, the concern is that people may switch from a more effective to a less effective method. Our data show that this was rarely the case; in fact, the introduction of implants (and reintroduction of IUDs) led to a substantial and sustained increase in the number of new users of all methods, including highly effective LARC methods. Our experience supports previous research demonstrating that use of modern contraception increases when more methods become available. Further, it suggests that the introduction of these new methods, and the concomitant improvement in service availability and quality, resulted in an acute increase in satisfied demand.^4^^,^^26^

Introduction of implants (and reintroduction of IUDs) led to a significant and sustained increase in the number of new users of all methods.

The literature, in particular Sullivan and colleagues’ work on method mix, skew, and its relationship to choice (and satisfaction),^14^ helped us think critically about the skew that we saw early in our project and to ask important questions about factors underlying clients’ choice of methods. While our goal was not to achieve an “ideal” method mix in which all methods have equal numbers of users, we did note a more equally distributed mix of methods from January to June 2015, including a very robust uptake of IUDs in DRC, which continues to the current months of programming.

The importance of a comprehensive program that includes both supply- and demand-side interventions cannot be emphasized enough. Good-quality training and coaching, excellent counseling, a consistent stock of contraceptives, and a welcoming clinic environment are necessary elements of the supply side of family planning services. Another key finding was that simply increasing knowledge and awareness was not enough. Barriers to access had to be addressed as well. Women wanted highly effective modern contraception. Identifying, exploring, and challenging social barriers to women getting those services, in particular, with influential and powerful leaders—religious leaders and police, for example—was instrumental in breaking down those barriers. We also found that investing time and effort in monitoring alongside district managers, providers, and community members and analyzing data together to make decisions and plan actions has been crucial to our success. In addition, conversations with both providers and clients helped us improve our strategies to increase access to a broader range of methods. We were able to make important program modifications to increase the quality of services and the acceptability of a broad range of methods. We focused on addressing the provider issues, particularly strengthening clinical skills and, importantly, counseling skills. We also adjusted our community communication strategies, so that both efforts reinforced the availability of a full range of methods and ensured that, regardless of method mix patterns, women were receiving the method of their choice and being provided the services and support to do so.

Simply increasing knowledge and awareness was not enough. Barriers to access had to be addressed as well.

Attention to method mix will continue to be a priority in our program, and we will make the analysis of method mix, using the 50% rule, a regular feature of our data for decision-making process. We will also explore using a recently developed tool called “average deviation” of method mix to deepen our understanding of the factors influencing method distribution.^27^ Given our success in increasing access to and use of LARCs, as suggested by the comparison with the recent national health survey data cited in this article, we will build on our experiences and increase our efforts in future programming to improve access to and use of permanent methods, which have been largely neglected in central African contexts.

Attention to method mix will continue to be a priority in our program.

## Supplementary Material

supplementary material

## Notes

**Peer Reviewed**
