# Supplementary material for: Rapid Contraceptive Uptake and Changing Method Mix With High Use of Long-Acting Reversible Contraceptives in Crisis-Affected Populations in Chad and the Democratic Republic of the Congo
Source: Glob Health Sci Pract. 2016 Aug 11;4(Suppl 2):S5–S20. doi: 10.9745/GHSP-D-15-00315 (PMC4990162; doi:10.9745/GHSP-D-15-00315)
Supplement: supplementary material [file GHSP-D-15-00315_index.html]

Supplement to Rapid Contraceptive Uptake and Changing Method Mix With High Use of Long-Acting Reversible Contraceptives in Crisis-Affected Populations in Chad and the Democratic Republic of the Congo | Global Health: Science and Practice

## GHSP-D-15-00315 Supplementary Material

Rattan et al. doi: 10.9745/GHSP-D-15-00315

- supplementary material - Rattan et al. doi: 10.9745/GHSP-D-15-00315
