# Supplementary material for: Rapid Contraceptive Uptake and Changing Method Mix With High Use of Long-Acting Reversible Contraceptives in Crisis-Affected Populations in Chad and the Democratic Republic of the Congo
Source: Glob Health Sci Pract. 2016 Aug 11;4(Suppl 2):S5–S20. doi: 10.9745/GHSP-D-15-00315 (PMC4990162; doi:10.9745/GHSP-D-15-00315)
Supplement: supplementary material [file 15-00315-Rattan-Supplementary-material.pdf]

Rattan J, Noznesky E, Hwang S, Galavotti C, Curry DW, Rodriguez M. Rapid contraceptive uptake and changing method mix with high use of long-acting reversible contraceptives in crisis-affected populations in Chad and the Democratic Republic of the Congo. Glob Health Sci Pract. 2016;4 Suppl 2:S5-S20. http://dx.doi.org/10.9745/GHSP-D-15-00315

SUPPLEMENTARY MATERIAL. SAFPAC Monitoring and Evaluation Data Flowchart

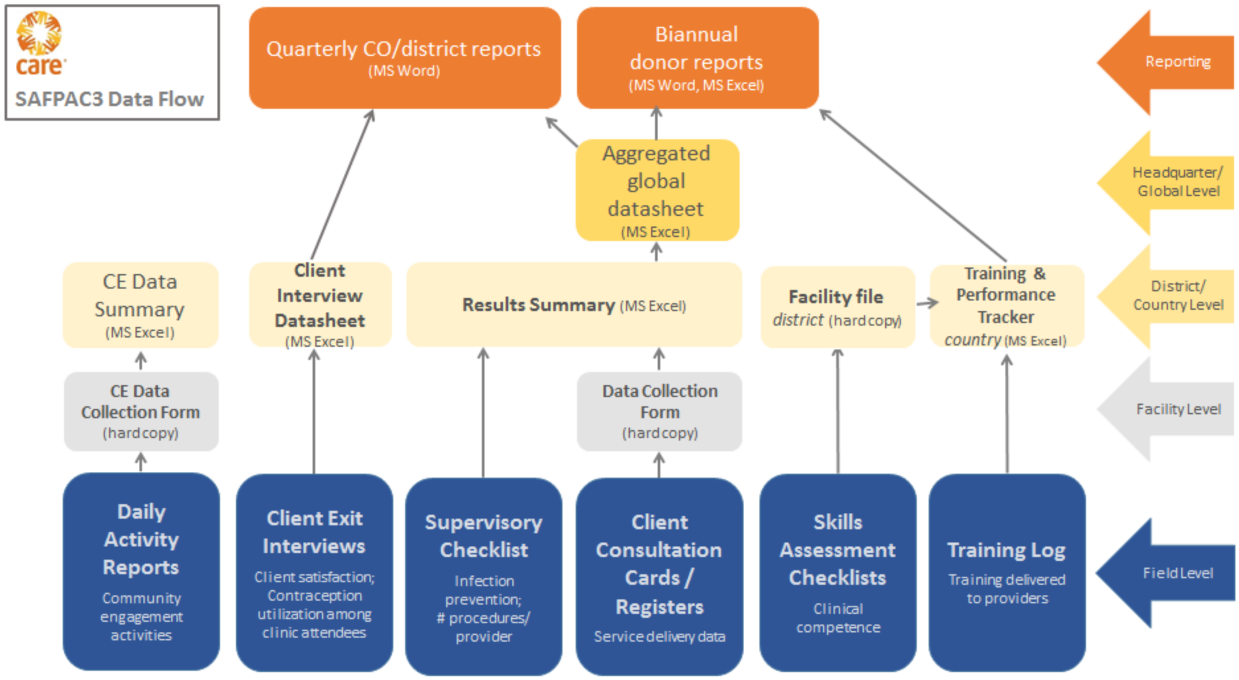

Abbreviations: CO, country office; CE, community engagement; SAFPAC, Supporting Access to Family Planning and Post-Abortion Care.

* To learn more about these tools, visit: http://familyplanning.care2share.wikispaces.net/SAFPAC+Monitoring+%26+Evaluation.
